# Inkjet Printing of Synthesized Melanin Nanoparticles as a Biocompatible Matrix for Pharmacologic Agents

**DOI:** 10.3390/nano10091840

**Published:** 2020-09-15

**Authors:** Matthew Ballard, Ashkan Shafiee, Elinor Grage, Max DeMarco, Anthony Atala, Elham Ghadiri

**Affiliations:** 1 Department of Chemistry, Wake Forest University, Winston-Salem, NC 27109, USA; ballmf17@wfu.edu (M.B.); gragec18@wfu.edu (E.G.); demamc18@wfu.edu (M.D.); 2 Wake Forest Institute for Regenerative Medicine, Wake Forest School of Medicine, Winston-Salem, NC 27157, USA; ashafiee@wakehealth.edu (A.S.); aatala@wakehealth.edu (A.A.); 3 Center for Functional Materials, Wake Forest University, Winston-Salem, NC 27109, USA; 4 Comprehensive Cancer Center, Wake Forest School of Medicine, Winston-Salem, NC 27157, USA

**Keywords:** synthesized melanin nanoparticles, drug release system, biocompatible electronics, inkjet printing

## Abstract

Melanin is a natural biopigment that is produced by melanocytes and can be found in most living organisms. The unique physical and chemical properties of melanin render it potentially useful for numerous applications, particularly those in which a biocompatible functional material is required. Herein, we introduce one important technology in which melanin can be utilized: a drug delivery system in terms of a biocompatible matrix. However, extracting melanin from different biological sources is costly and time-consuming and introduces variabilities in terms of chemical structure, properties, and functions. Hence, a functionally reproducible system is hard to achieve using biologically extracted melanin. Here we report the synthesis of melanin nanoparticles of controlled uniform sizes and chemical characteristics. The optical, chemical, and structural characteristics of synthesized nanoparticles were characterized by optical confocal photoluminescence (PL) imaging, scanning electron microscopy (SEM), Fourier transform infrared spectroscopy (FTIR), and Zeta potentiometry. The melanin nanoparticles have 100 nm size and a narrow size distribution. The advantage of a nanoparticle structure is its enhanced surface-to-volume ratio compared to bulk pigments, which is important for applications in which controlling the microscopic surface area is essential. Using the inkjet printing technique, we developed melanin thin films with minimum ink waste and loaded them with methylene blue (our representative drug) to test the drug-loading ability of the melanin nanoparticles. Inkjet printing allowed us to create smooth uniform films with precise deposition and minimum ink-waste. The spectroscopic analysis confirmed the attachment of the “drug” onto the melanin nanoparticles as a matrix. Hence, our data identify melanin as a material system to integrate into drug release applications.

## 1. Introduction

Melanin, one of the most ubiquitous and stable biopolymers, is produced in living organisms by a particular group of cells called melanocytes [1,2,3]. It is found in the organs of almost every higher-level organism, including human skin [4], hair [5], eyes [6], and the brain [7]. And occurs in different forms including eumelanin (a black-brownish pigment), pheomelanin (a red pigment), and neuromelanin (which is generated by dopaminergic neurons in the brain) [1,2,7]. Its functionality is defined (although not fully understood) by its physical and chemical properties, such as its featureless broad optical absorption, antioxidant properties, and free radical scavenging behavior [1,2,5]. Its functional and photochemical properties are yet to be explored [8,9,10].

Fabricating bioengineered devices for in vivo applications, including drug release systems, requires biocompatible materials [11,12]. There is a global need to identify materials that possess functional properties for special applications and that also have biocompatible characteristics suitable for use in cellular environments [13]. Most of the studies surrounding melanin deal with characterizing its structural and chemical properties [3,5,9,10,14]. The biocompatibility of melanin is unambiguous. The biocompatibility of commercially available melanin pigments has been previously investigated by incorporating melanin pigments in the vicinity of the cells and the study of cell survival [15]. As a natural biopigment, its biocompatibility makes it a suitable candidate for the fabrication of medical devices that can be used both in vitro and in vivo. Although this pigment offers great potentials, the field of medical device fabrication using melanin is relatively young and hardly explored. Nevertheless, the chemical composition and characteristics of melanin pigments extracted from biological sources often vary by the nature of the source, which complicates determining the pigment’s characteristics accurately [2,8,16]. The chemical synthesis approach and post synthesis treatment also would strongly affect the physical, structural and chemical properties of the melanin product. Therefore, by the commercially available melanin and the melanin’s extracted from biological sources, it is hard to examine a reproducible functionality (when it comes to integrating melanin for specific applications). Therefore, a reliable and reproducible protocol for the synthesis of melanin may improve the biofabrication process and enable melanin use for different biomedical engineering-related purposes.

Additive manufacturing, specifically printing technologies, have been employed in many different applications ranging from electronic device fabrication [17,18], including transistors [19], light emitting diodes [20], and solar cells [21] to biofabrication [22,23,24,25], tissue engineering [26,27,28], and optimizing the surgical planning [29]. For example, in drug delivery systems, inkjet printing can provide a platform with which to engineer the surface properties of the host material in order to obtain zero-order release kinetics [30]. This includes preparing a homogeneous distribution of materials throughout the surface or creating various diffusion gradients at different sites of the device [30,31,32]. The computer-controlled fabrication processes of printers, deposition of homogeneous films with high resolution and reproducibility, as well as the low amount of waste generated, single them out among all other fabrication techniques [30,31]. An exact amount of the required material is dispensed onto the precise location of the substrate to construct a favorably specific release profile that may not be possible otherwise [33]. As such, printers provide a unique platform to deposit expensive materials (ink) with high precision and low cost [34].

Various drug delivery techniques involve a matrix that hosts the drug and releases it, once needed, using different cues such as thermal or electrical stimulators [35]. In this case, pharmacologic agents can be loaded into matrices, such as polycaprolactone, poly (3,4-ethylenedioxythiophene) polystyrene sulfonate, and polyethylene oxide, to be stored and released when needed [30,36]. The characteristics of these matrices can affect the release efficacy dramatically [37]. A number of functional materials are being investigated to enhance drug delivery outcomes. In drug delivery systems with matrices, it is common to have a time-dependent release; as such, the released amount is a function of time wherein the release is maximal at the initial stage and decreases gradually [38]. This must be addressed to maintain the drug load within an appropriate therapeutic window for an extended duration. Several researchers have been working to maintain the drug release within a therapeutic window by using different methods, such as employing low-permeability materials to reduce the initial release and obtain zero-order release kinetics [30]. We previously used a “pixel-based drug release” system that consists of several miniature drug cartridges; by optimizing the release from each miniature drug cartridge (pixel) and using a cascade of release from individual pixels in a time-lag fashion, one can maintain the drug within the therapeutic window [36].

Herein, we report the synthesis of melanin nanoparticles with controlled uniform size, shape, and chemical composition using synthetic nanochemistry. The structural and physical properties of the synthetic melanin nanoparticles were investigated and compared to those of natural melanin known as sepia melanin that were extracted from biological sources. Using the inkjet method, melanin nanoparticles were printed to obtain homogeneous thin films in the form of a matrix with minimum ink waste. Melanin can be considered a potential biocompatible material for matrix-based drug delivery. We investigated the potential of synthesized melanin nanoparticles with a controlled size and chemical structure for drug loading. The inkjet-printed melanin nanoparticles were then loaded with methylene blue (MB), and the loaded drug was tested using optical spectroscopy. The increased surface-to-volume ratio of nanoparticles compared to bulk pigments aimed to provide an increased surface area for drug loading. We used MB as a model drug, this agent is mainly used to treat severe methemoglobinemia (a disease in which the methemoglobin blood level is elevated).

## 2. Materials and Methods

### 2.1. Preparation of Melanin Nanoparticles

We synthesized melanin-based nanomaterials using nanochemistry-based approaches that allow for controlling the size and size distributions of the nanoparticles. The melanin nanoparticles were prepared using the oxidative polymerization of the organic monomeric units, which is similar to the biological mechanism of melanin formation by melanocytes in live organisms [2,3]. The monomeric units of the melanin differ depending on their chemical compositions, in the case of eumelanin, the monomeric units were 5,6-dihydroxyindole and 5,6-dihydroxyindole-2-carboxylic acid. The synthetic melanin nanoparticles in this study are prepared from dopamine hydrochloride (Sigma-Aldrich, St. Luis, MO, USA) as the starting precursor. The natural pigments (sepia melanin) are extracted from biological sources [2]. By adjusting synthetic parameters such as pH, temperature, and reaction time, the chemical compositions and sizes of the nanoparticles were controlled. The nanoparticles were separated from the solution batch by centrifugation (Beckman Coulter, Indianapolis, IN, USA) at 18,000 RPM 3 to 4 times. Melanin nanoparticle ink was prepared using deionized (DI) water as a solvent. This procedure allows synthesizing different melanin types with changing the precursor as the starting material. 

### 2.2. Inkjet Printing of Melanin Nanoparticles

**Figure 1:**
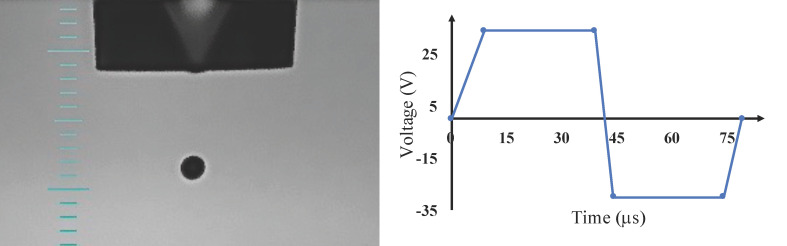
Inkjet printing of synthesized melanin nanoparticles. (**a**) An 80 μm nozzle was used, and perfect spherical droplets without satellites were printed with 0.35 m/s and 70.65 μm for drop velocity and diameter, respectively. (**b**) The voltage wave applied to the piezoelectric crystal of the printer. The Rise time 1 was set as 9 μs, it remained 30 μs in Dwell voltage (34 V), in 5 μs fell to reach Echo voltage (−30 V) and stayed in Echo voltage for 30 μs and rose back to the Ideal voltage (0 V) in 5 μs.

To integrate the synthetic melanin nanoparticles, we used different thin film deposition techniques such as simple drop-casting as well as a more advanced inkjet printing method. The most critical task in substrate preparation is to optimize the surface energy to control the wettability of the substrate with inkjet printed materials. In this case, we treated the surface with a plasma cleaner PDC-32G (Harrick Plasma, Ithaca, NY, USA) for 35 s with medium power. Plasma cleaning altered the energy of the surface that helps the inkjet-printed films spread across the surface. The prepared melanin nanoparticle ink was printed using a piezoelectric inkjet printer (Jetlab IX, Microfab Technologies, Plano, TX, USA). To optimize the ink for printing, the freshly prepared melanin nanoparticles were centrifuged and dispersed at different concentrations in water. The optimum printable ink was prepared using melanin nanoparticles with a concentration 37.5 μg/mL in DI water. Prior to the printing process, the ink was sonicated for 2 min to avoid aggregation of particles and to prevent nozzle clogging. An 80 μm nozzle was used, and in order to make perfect droplets without any satellite, the printing parameters were adjusted. Satellites or droplets’ tails may dissociate from the main droplet and form smaller daughter drops that can reduce the quality of thin films. The optimized printing parameters were found as 9, 30, 5, 30, and 5 μs for Rise time 1, Dwell time, Fall time, Echo time, and Rise time 2. In addition, the Idle, Dwell, and Echo voltages were set as 0, 34, and −30 V. This configuration provided droplets with 0.35 m/s, 184 pl, and 70.65 μm for drop velocity, volume, and diameter, respectively. Figure 1 demonstrates the drop on fly, and voltage standard wave applied on the piezoelectric crystal in the inkjet nozzle. Glass substrates were used and 1 cm^2^ films were printed. The inkjet-printed films were thermally treated at 60 °C for one hour to evaporate the solvent.

## 3. Results and Discussion

### 3.1. Characterization of Melanin Nanoparticles and Films

**Figure 2:**
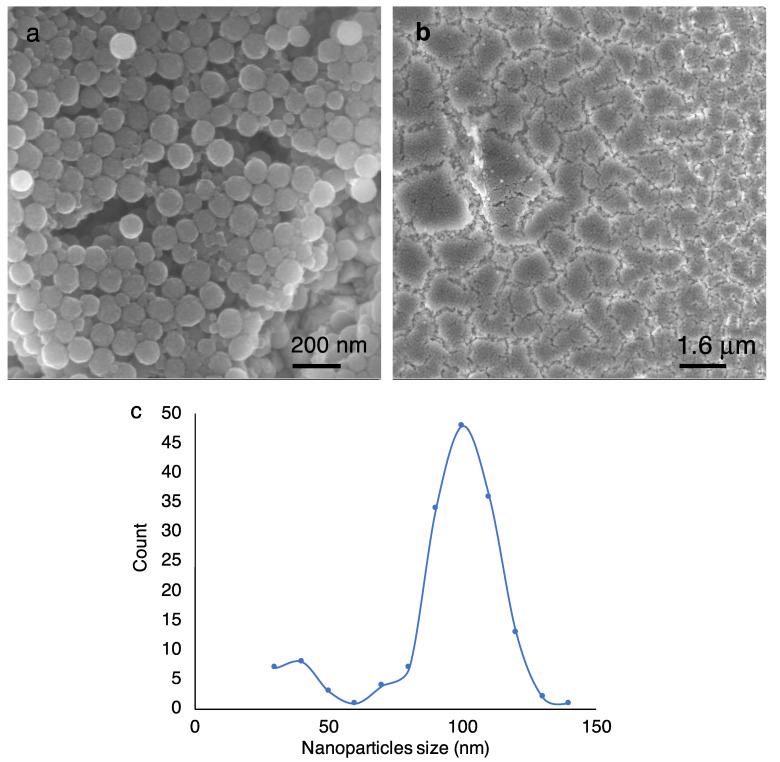
Scanning electron microscopy (SEM) images of synthetic melanin nanoparticles. (**a**) Synthetic nanoparticles ink. (**b**) Inkjet-deposited melanin nanoparticle-based thin film. Both images show uniform-size melanin nanoparticles of 100 nm. The large-scale image shows a uniform coating. Nanoparticles are not deformed or agglomerated during the ink preparation and printing process. (**c**) Nanoparticle size distribution analysis using SEM images shows a peak at 100 nm with full width half maximum (FWHM) of 26 nm, and less than 5% of the particles being 40 nm.

**Scanning Electron Microscopy** (**SEM**) **analysis of the melanin nanoparticles and inkjet-printed films.** Figure 2 shows the SEM images of melanin nanoparticles and inkjet-printed film. The SEM images were acquired using a (Zeiss GeminiSEM, Oberkochen, Germany, 300 high-resolution field effect SEM). Synthetic melanin nanoparticles were of a relatively small particle size (100 nm) and had a narrow size distribution, as it is shown in Figure 2c. The full width half maximum (FWHM) of the broadening of the size distribution is 26 nm. Some small fraction of nanoparticles (less than 5%) have a smaller size of 40 nm. The smaller particles can be separated by centrifugation at a speed higher than 18,000 RPM that is applied here. It should be noted that to identify the size distribution, often dynamic light scattering (DLS) is used. However, DLS is more sensitive to big particles, and if agglomeration has formed in the sample, it will be assigned to the presence of big nanoparticles [39]. Therefore, the SEM analysis provides more detailed and reliable data in that regard. SEM of the films also confirmed a uniform close-packed layer made of uniform-size spherical nanoparticles that were not deformed or agglomerated during the ink preparation and printing process. The post-printing process and film heat treatment resulted in evaporated solvent in the film, which left microscopic cracks. The microscopic surface area, which defines the available surface area for chemicals that are adsorbed into the film, is an important parameter for many applications, including drug loading [40]. Therefore, the existence of such cracks could enhance the microscopic surface area and help increase the amount of loaded drugs. It has been previously reported by our group and others that by increasing the microscopic surface area the dye molecule absorption (herein methylene blue) is enhanced [41,42]. This must be optimized in device fabrication to get a balance between loaded drug and release performance. 

The pH of the nanoparticle solution is 7.3. The surface potential of the nanoparticles measured by the Zeta potential analyzer (using Zetasizer Brookhaven Instrument, Holtsville, NY, USA) is −41.63 mV that confirms the stability of the nanoparticles in solution. The magnitude of the Zeta potential indicates the stability of nanoparticles against aggregation [43]. In other words, nanoparticles with any zeta potential out of −30 to +30 mV possess the appropriate electrostatic repulsive force to prevent aggregation. 

**Figure 3:**
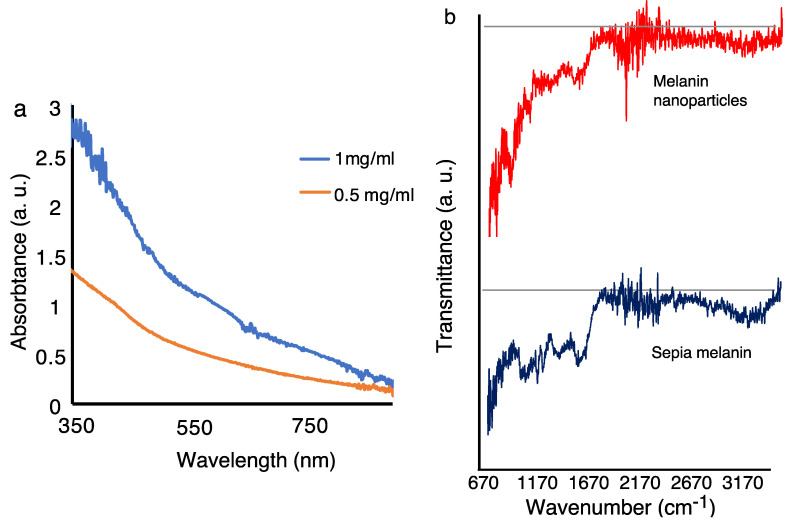
Ultraviolet-Visible (UV-Vis) and attenuated total reflectance-Fourier transform infrared spectroscopy (ATR FTIR) characterization of synthetic melanin nanoparticles and sepia melanin. (**a**) The optical absorption spectrum of nanoparticles shows a featureless broadband absorption from UV to near IR. (**b**) ATR FTIR transmission spectrum of synthetic melanin nanoparticles and sepia (natural) melanin. The ATR FTIR spectra of synthetic melanin nanoparticles and sepia melanin show a high degree of similarity. Both spectra show broad absorption around 3170 cm^−1^, which can be due to associated or polymeric hydroxy groups. The fingerprint regions between 700 cm^−1^ and 1500 cm^−1^ resemble each other, indicating that the functional groups are similar in both melanin types.

**Linear optical and Fourier transform infrared spectroscopy (FTIR) of melanin.** To characterize the optical characteristics of melanin nanoparticles, ultraviolet (UV)-Vis-near-infrared (NIR) optical absorption and photoluminescence (PL) spectroscopy were performed. The UV-Vis optical absorption of the melanin nanoparticles (Figure 3a) shows the characteristic featureless monotonic UV to NIR broadband absorption with more UV-extended absorption, which is consistent with the well-known optical characteristics of the natural eumelanin. A linear increase in the UV to NIR is observed by increasing the nanoparticles’ concentration in the solution. 

FTIR is used to characterize the samples by monitoring the vibrational signature of the melanin. The attenuated total reflectance FTIR (ATR FTIR) spectrum of synthetic melanin nanoparticles and sepia melanin (which is a standard melanin extracted from biological source [2,8]) are shown in Figure 3b. For FTIR measurements, the melanin nanoparticles were extracted and measured in the form of a dry pellet. Additionally, the sepia melanin was measured in the form of a powder pellet. The ATR FTIR spectra of synthetic melanin nanoparticles and sepia melanin show a high degree of similarity. Both spectra show broad absorption around 3170 cm^−1^, which can be due to associated or polymeric hydroxy groups. The fingerprint regions between 700 cm^−1^ and 1500 cm^−1^ resemble each other. These data indicate that the functional groups are almost identical in both melanin types. 

**Figure 4:**
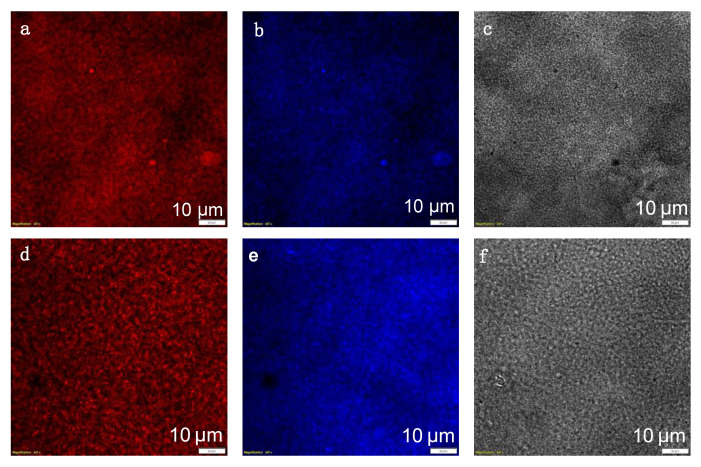
Confocal photoluminescence (PL) imaging of melanin nanoparticles with excitations and PL recorded at different spectral regions. The excitation and detection wavelength regions are respectively (**a**) 561 nm, 570–620 nm; (**b**) 561 nm, 621–721 nm; (**d**) 640 nm, 570–620 nm; (**e**) 640 nm, 621–721 nm. (**c**,**f**) confocal microscopic images obtained with the 561 nm and 640 nm, respectively. The confocal images are correlated with the PL images. The small dots represent melanin nanoparticles.

**Linear confocal PL imaging of melanin samples.** The nature of broadband absorption in melanin is not entirely understood, it is unclear whether it is all due to electronic transitions or is partially influenced by optical features such as light scattering. The featureless broadband absorption of melanin makes its use for linear optical absorption spectroscopy for detailed structural characterizations challenging. Therefore, we integrated PL spectroscopy/microscopy (Olympus PL Confocal Microscope, FV3000, Tokyo, Japan) for more sophisticated analysis. For PL microscopy (Figure 4), melanin nanoparticles are immobilized in agarose hydrogel as an optically transparent matrix. This allowed for creating homogeneous samples with a thickness of a few hundred micrometers with no interference by the matrix with the optical imaging. The PL images (Figure 4a,b,d,e) and confocal images (Figure 4c,f) demonstrate a film of small nanoparticles that is homogeneous over a large scale of more than 100 μm. Two different wavelengths of 561 and 640 nm were used to excite the samples, and the PL spectra were measured at two different wavelength regions of 570–620 nm (shown in false red) and 621–721 nm (shown in false blue). Figure 4a,b represent the PL image upon excitation at 561 nm, while Figure 4d,e represent the PL upon excitation at 640 nm. Figure 4c,f are confocal images of nanoparticles recorded for each measurement. 

**Figure 5:**
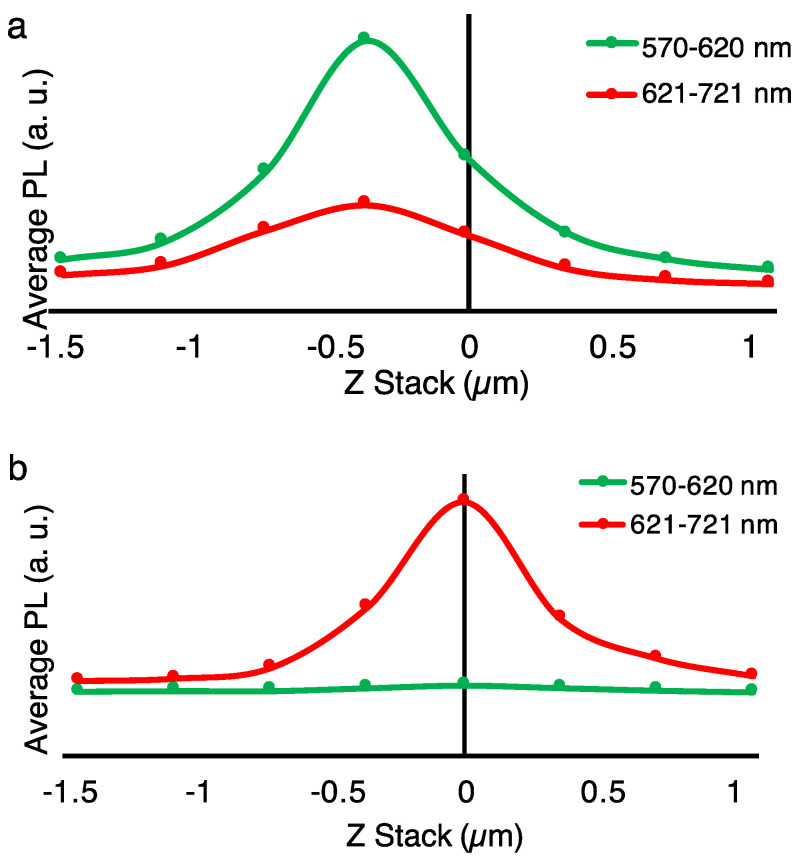
Photoluminescence (PL) measurements at different excitations and PL detection regions for melanin nanoparticles with a size of 100 nm. The sample is composed of a several-micrometer-thick film of nanoparticles deposited on a glass substrate. The y-axis represents the PL intensity, while the x-axis represents the z-stack, with the zero being in the middle of the sample. The excitation and detection wavelength regions are (**a**) 561 nm, 570–620 nm; 561 nm, 621–721 nm and (**b**) 640 nm, 570–620 nm; 640 nm, 621–721 nm. The amplitude of the PL is enhanced when the PL detection window is close to the excitation wavelength. This result suggests that the broadband optical absorption in melanin is based on a superposition of multiple chromophoric units; by applying different excitations, we can selectively excite different units. This result helps to unravel the nature of broadband ultraviolet to near-infrared absorption in melanin pigments.

Interestingly the PL spectra (Figure 5) correlate with the excitation wavelength; when the excitation was at 561 nm, the maximum PL was at the shorter wavelengths of 570–620 nm, whereas an excitation at 640 nm elicited a maximum PL at the longer wavelengths of 621–721 nm. It should be noted that the intensity of the exciting beam was equal in both measurements. This result indicates that the optical absorption/emission of the melanin is a superposition of the multiple chromophoric units’ absorption/emission. This performance resembles the optoelectronic behavior of the organic conjugated polymeric systems in which they, in contrast to inorganic semiconductors, do not have a single band-edge. This analysis is particularly important to consider for the integration of melanin nanoparticles in biomedical applications that rely on the PL and optoelectronic characteristics.

### 3.2. Melanin Nanoparticles for Drug Loading

**Scheme 1:**
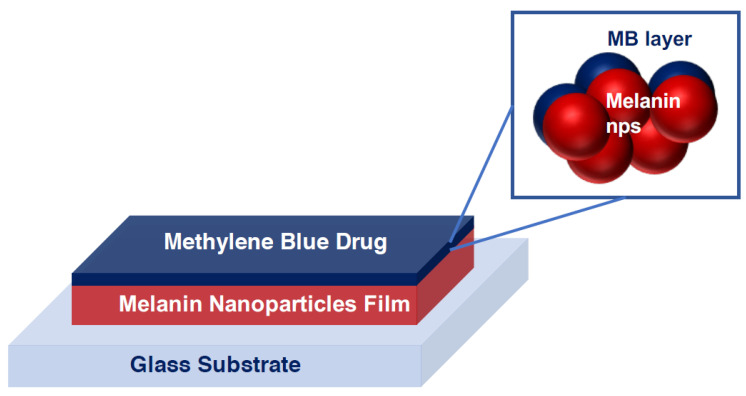
The structure of the fabricated drug loading device based on the inkjet-printed melanin nanoparticle thin film on glass substrates. The MB is loaded on melanin nanoparticles as a test model drug.

We integrated the melanin nanoparticles as a biocompatible organic host for drug loading. The biocompatibility of the melanin pigments from commercially available sources has been previously investigated and well documented. Herein, we integrated the synthesized nanoparticles with controlled chemical composition and morphology as a reproducible melanin source in drug loading studies for target drug release applications. It is well known that the surface-to-volume ratio is increased when pigments are in the nanometer scale. This increases the microscopic surface area, which helps to control the amount of the drug loaded onto the film. The homogeneity in particle size and within the film area is also beneficial for a controlled load-and-release behavior. Our model drug (MB) was printed into the melanin films; after drying the sample in the air for a few minutes, the samples were rinsed with deionized water to remove any excess dye. Scheme 1 shows the device structure for the drug-loading experiments. The melanin nanoparticles-based film is prepared by inkjet printing of the nanoparticle solution. The macroscopic area of the film can be adjusted by the inkjet printing. The highest resolution in inkjet printing is few micrometers. The macroscopic surface area is typically 1 cm^2^ in our drug loading experiments. 

**Figure 6:**
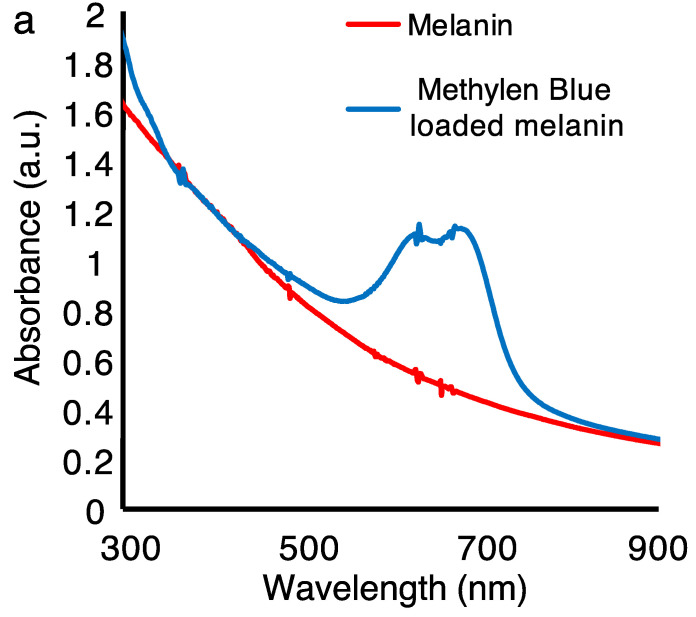

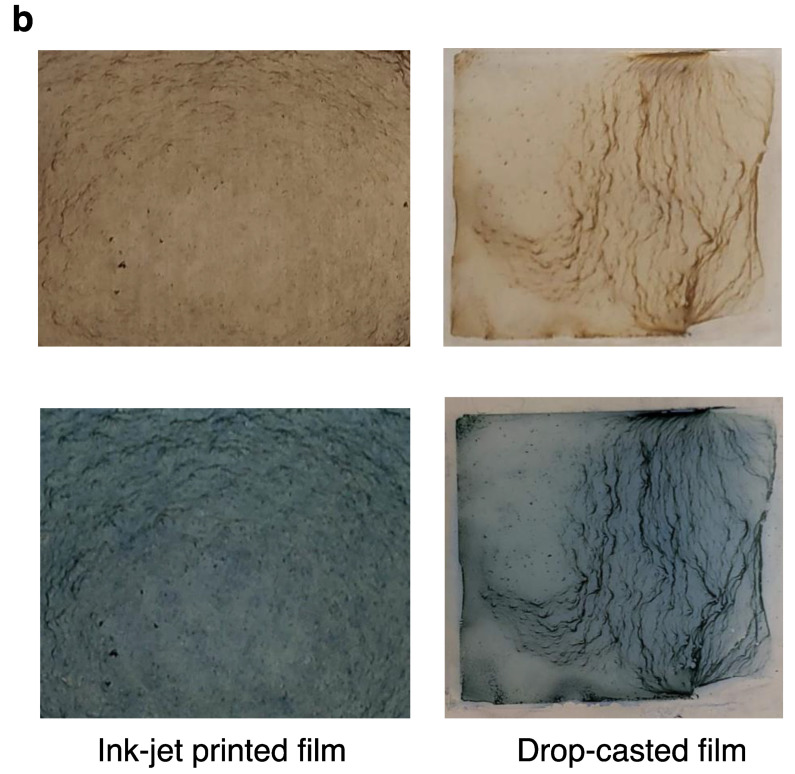
Characteristics of the melanin nanoparticles-based films prepared by inkjet printing and drop-casting before and after drug loading. (**a**) Ultraviolet to near-infrared optical absorption of the melanin nanoparticles films before and after loading with the drug. We used methylene blue (MB) as a model drug. The characteristic absorption peaks of the MB at 618 nm and 670 nm superposed on the broadband absorption of the melanin confirm the successful loading of the drug. (**b**) The photograph of the films also clearly confirms the drug loading. The melanin films are characterized by a brownish color while the MB-loaded melanin films appear bluish. The quality of thin films prepared by the inkjet printing technique impacts the fabrication of a reliable drug delivery system that requires a homogeneous deposition method. As is shown, the inkjet-printed melanin and MB-loaded melanin films are clearly more homogenous than those prepared by other techniques.

Figure 6a shows the UV-NIR optical absorption of the melanin nanoparticles films before and after treating them with the drug. The characteristic absorption peaks of the MB at 618 nm and 670 nm were superposed on the broadband absorption of the melanin, confirming the successful loading of the drug. Based on the height of the optical absorption peak, the amount of MB was estimated to be 0.29 μmol (within the irradiation beam size in the optical measurement). The melanin films were characterized by a brownish color while the MB loaded melanin films appeared bluish. Importantly, the quality of thin films prepared using the inkjet printing technique can impact the fabrication of a reliable drug delivery system, and we previously showed how to optimize and predict the amount of drug release to enhance the accuracy and maximum therapeutic efficacy [36]. To that end, one critical factor when fabricating a predictable and reliable drug delivery system is to consistently distribute the agent on the host layer; this necessitates a homogeneous deposition. As is depicted in Figure 6b, inkjet-printed melanin films are more homogenous than films prepared by other techniques. 

The biocompatibility of melanin nanoparticles, as well as their ability to host drugs on a surface position them as a potential matrix for drug delivery systems that may be aimed for use in the vicinity of cells and tissues. 

## 4. Conclusions

For many years, the primary function of melanin in biological systems was understood only as a photoprotectant, as it strongly absorbs UV photons. Herein, we demonstrate new potentials for the integration of this biocompatible pigment for medicinal applications, including the drug release system. We synthesized uniform-sized melanin nanoparticles and prepared a printable ink. The nanoparticle solution was dispersed in water, which was then used to prepare uniform thin films using the inkjet printing method. The ATR FTIR spectra of synthetic melanin nanoparticles and sepia melanin showed a high degree of similarity. Both spectra showed broad absorption around 3170 cm^−1^, which could be due to the associated or polymeric hydroxy groups. The fingerprint regions between 700 and 1500 cm^−1^ resembled each other, indicating that the functional groups were almost identical in both melanin types. The PL images and spectra of the melanin nanoparticles correlated with the excitation wavelength; when the excitation was at 561 nm, the maximum PL was at the shorter wavelengths of 570–620 nm, whereas an excitation of 640 nm elicited a maximum PL at the longer wavelengths of 621–721 nm (the excitation beams were of similar intensities in both measurements). This result indicates that the optical absorption/emission of the melanin is a superposition of the multiple chromophoric units’ absorption/emission, which resembled the optoelectronic behavior of the organic conjugated polymeric systems. The melanin nanoparticle-based films were used as a biocompatible matrix for drug-loading applications. Synthetic melanin nanoparticles were of a relatively uniform and small particle size of 100 ± 13 nm. The nanometer size range would increase the surface-to-volume ratio (increasing the microscopic surface area for drug loading), and the homogeneities in particle size and within the film area are also beneficial for a controlled load-and-release mechanism. The surface potential of the nanoparticles measured by the Zeta potential analyzer is −41.63 mV that confirms the stability of the nanoparticles ink. The optical absorption spectrum clearly confirmed the loading of MB within the inkjet-printed melanin films. The quality of thin films prepared by the inkjet printing technique can impact the fabrication of a reliable drug delivery system. The inkjet-printed melanin films are more homogenous than those prepared using other techniques.
